# Polymorphisms of the Ovine *BMPR-IB*, *BMP-15* and *FSHR* and Their Associations with Litter Size in Two Chinese Indigenous Sheep Breeds

**DOI:** 10.3390/ijms160511385

**Published:** 2015-05-18

**Authors:** Weimin Wang, Shijia Liu, Fadi Li, Xiangyu Pan, Chong Li, Xiaoxue Zhang, Youji Ma, Yongfu La, Rui Xi, Tingfu Li

**Affiliations:** 1 College of Animal Science and Technology, Gansu Agriculture University, Lanzhou 730000, China; E-Mails: wangwm@gsau.edu.cn (W.W.); 15604258787@163.com (S.L.); bendanpanxiangyu@163.com (X.P.); lichong@gsau.edu.cn (C.L.); zhangxx@gsau.edu.cn (X.Z.); yjma@gsau.edu.cn (Y.M.); 15294158469@163.com (Y.L.); 527233669xr@sina.com (R.X.); 2 Engineering Laboratory of Sheep Breeding and Reproduction Biotechnology in Gansu Province, Minqin 733300, China; E-Mail: litingfu@ztyangye.com; 3 Minqin Zhongtian Sheep Industry Co., Ltd., Minqin 733300, China

**Keywords:** *BMPR-IB* gene, *BMP-15* gene, *FSHR* gene, litter size, Small Tailed Han sheep, Hu sheep

## Abstract

The Small Tailed Han sheep and Hu sheep are two prolific local sheep in China. In this study, the polymorphisms of *BMPR-IB* (Bone morphogenetic protein receptor IB), *BMP-15* (Bone morphogenetic protein 15) and *FSHR* (follicle stimulating hormone receptor) were investigated to check whether they are associated with litter size in Small Tailed Han sheep and Hu sheep. Consequently, three polymorphisms, *FecB* mutation in *BMPR-IB* (c.746A>G), *FecG* mutation in *BMP-15* (c.718C>T) and the mutation (g. 47C>T) in *FSHR* were found in the above two sheep breeds with a total number of 1630 individuals. The single marker association analysis showed that the three mutations were significantly associated with litter size. The ewes with genotype *FecB^B^*/*FecB^B^* and *FecB^B^*/*FecB^+^* had 0.78 and 0.58 more lambs (*p* < 0.01) than those with genotype *FecB^+^*/*FecB^+^*, respectively. The heterozygous Han and Hu ewes with *FecX^G^*/*FecX^+^* genotype showed 0.30 (*p* = 0.05) more lambs than those with the *FecX^+^*/*FecX^+^* genotype. For *FSHR* gene, the ewes with genotype *CC* had 0.52 (*p* < 0.01) and 0.75 (*p* < 0.01) more lambs than those with genotypes *TC* and *TT*, respectively. Combined effect analyses indicated an extremely significant interaction (*p* < 0.01) between the random combinations of *BMPR-IB*, *BMP-15* and *FSHR* genes on litter size. In addition, the Han and Hu ewes with *BB/G+/CC* genotype harbor the highest litter size among ewes analyzed in current study. In conclusion, *BMPR-IB*, *BMP-15* and *FSHR* polymorphisms could be used as genetic markers in multi-gene pyramiding for improving litter size in sheep husbandry.

## 1. Introduction

The *FecB* (Fec = Fecundity, B = Booroola) mutation plays a vital role in increasing ovulation rate and prolificacy in ewes. This mutation (c.746A>G) was in *BMPR-IB* (Bone morphogenetic protein receptor IB) gene that located on chromosome 6,which was first found to be significantly associated with litter size in Booroola Merino ewes [1,2,3].

*BMP-15* (Bone morphogenetic protein 15) gene belongs to the *TGFβ* (Transforming growth factor-β) family, which acts as a key regulator of granulosa cell (GC) processes in ovarian follicular development [4,5]. The sheep *BMP-15* gene is located on the X chromosome [4]. The c.718C>T mutation (named *FecX^G^*; Galway mutation) in *BMP-15* gene was first identified in Cambridge and Belclare sheep, which increased ovulation rate and infertility [6].

*FSHR* (Follicle stimulating hormone receptor) gene was first identified in rat Sertoli cells and may have an influence on the FSH (follicle stimulating hormone) signal transduction [7]. Additionally, FSH has been reported to play an important role in the development of antral follicles [8,9]. A variety of mutations were found in the 5' flanking region of ovine *FSHR* gene, which were significantly associated with litter size in Australian sheep, Hu sheep and Small Tailed Han sheep [10,11,12].

The Small Tailed Han sheep and Hu sheep were originally raised in Shandong Province and Jiangsu Province, China [13]. They quickly gained the attention of Chinese sheep breeders and were largely used in the modern hybridization system as female parents because of their reputation for high fertility. To date, there are no reports about the combined effect of *BMPR-IB*, *BMP-15* and *FSHR* genes on litter size of Small Tailed Han sheep and Hu sheep. Therefore, the objectives of this study were to investigate the relationships of single nucleotide polymorphisms (SNPs) in *BMPR-IB*, *BMP-15* and *FSHR*, and their combined effect with litter size, which may serve as valuable markers for female fertility selection at the early stage in Small Tailed Han sheep and Hu sheep.

## 2. Results

### 2.1. Genotyping and Allele Frequency Analysis

**Figure 1:**
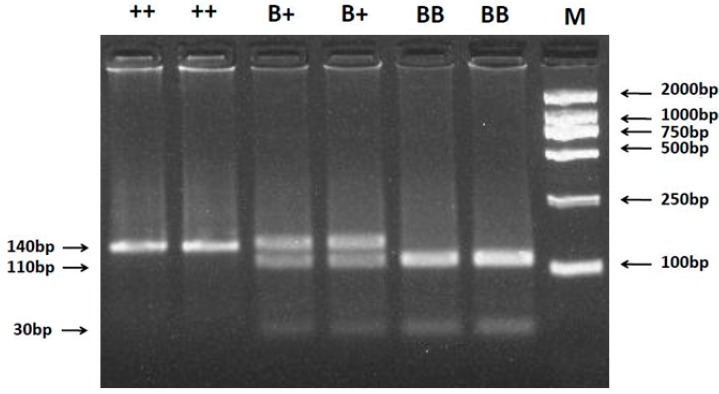
PCR-RFLP results of different genotypes of the PCR products digested by enzyme *Ava* IIc.746A>G of ovine *BMPR-IB* polymorphisms. The genotypes are marked on the top of the lanes. M: DNA Marker (DL2000).

A 140 bp PCR product containing the c.746A>G polymorphism in the coding region of *BMPR-IB* gene was digested using the *Ava* II restriction enzyme. The digestion generated three fragments, and the 140 bp, 110 bp + 30 bp and 140 bp + 110 bp +30 bp bands represented *++*, *BB* and *B+* genotypes, respectively (Figure 1).

**Figure 2:**
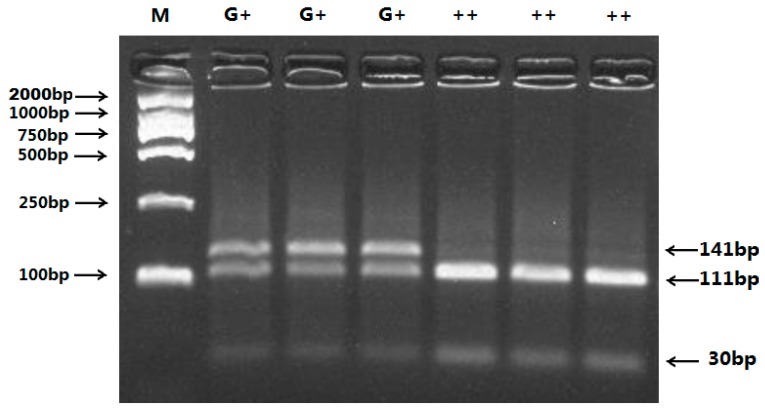
PCR-RFLP results of different genotypes of the PCR products digested by enzyme *Hinf* I c.718C>T of ovine *BMP-*15polymorphisms. The genotypes are marked on the top of the lanes. M: DNA Marker (DL2000).

Additionally, the 141 bp PCR product of the c.718C>T polymorphism of the *BMP-15* gene was digested with *Hinf* I, generating two fragments: the 141 bp + 111 bp + 30 bp and 111 bp + 30 bp bands represented *G+* and *++* genotypes, respectively (Figure 2). In ewes studied, none of them carried homozygous genotype (*GG*).

**Figure 3:**
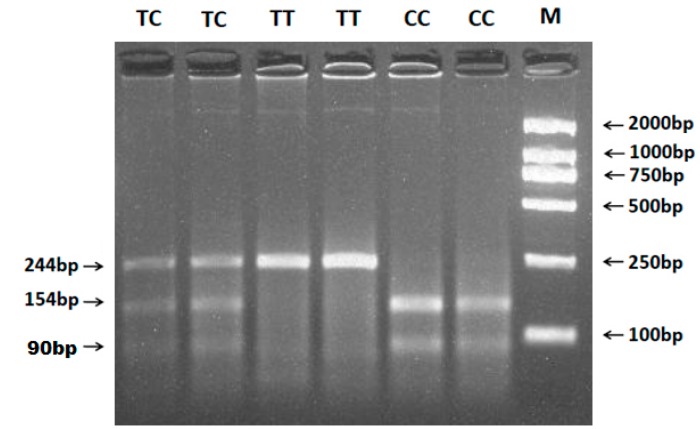
PCR-RFLP results of different genotypes of the PCR products digested by enzyme *BsiE* I g.47C>T of ovine *FSHR* polymorphisms. The genotypes are marked on the top of the lanes. M: DNA Marker (DL2000).

The 244 bp PCR product of the g.47C>T of the *FSHR* gene was digested by *BsiE* I and the result is shown in Figure 3. The fragment length of the *TT* genotype was 244 bp, while *CC* and *CT* genotypes were 154 bp + 90 bp and 244 bp + 154 bp + 90 bp, respectively.

The allele and genotype frequency were analyzed in the experimental populations (Table 1). All these three mutations were detected in both Small Tailed Han sheep and Hu sheep. The c.746A>G polymorphism of the *BMPR-IB* gene showed a higher frequency of allele G (B) than allele A (+), and the *GG* (*BB*) genotype was predominant in total population. The *CC* (*++*) genotype of the c.718C>T polymorphism of the *BMP-15* gene was higher in all population. Whereas, *TT* genotype of the g.47C>T polymorphism of *FSHR* gene was higher than the *CC* and *TC* genotypes in the population.

### 2.2. Single Marker-Trait Association

The least squares means and SE (standard error) for litter size of individuals with different genotypes in Small Tailed Han sheep and Hu sheep are shown in Table 2. The association analysis revealed that the three polymorphisms found in this study were significant, or tend to be associated with (*BMPR-IB*, *p* < 0.01; *BMP-15*, *p* = 0.05; and *FSHR*, *p* < 0.01) litter size in Hu and Han sheep.

Association analysis results indicated that the ewes with genotypes *BB* and *B+* had 0.78 (*p* < 0.01) and 0.58 (*p* < 0.01) gave birth to more lambs than those with genotype *++* in the experimental population, respectively, while the ewes with genotype *G+* had 0.30 (*p* = 0.05) had more lambs than the *++* genotype ones. The ewes carrying genotype *CC* had 0.52 (*p* < 0.01) and 0.75 (*p* < 0.01) had more lambs when compared with the ewes carrying genotypes *TC* and *TT*, respectively.

More specifically, the ewes with genotypes *BB* and *B+* had 0.85 (*p* < 0.01) and 0.57 (*p* < 0.01) more lambs than those with genotype *++* in Small Tailed Han sheep, respectively. The c.718C>T polymorphism of the *BMP-15* gene was not significantly associated with litter size in Small Tailed Han sheep (*p* > 0.05). The ewes carrying genotype *CC* and *TC*of the *FSHR* gene had 0.63 (*p* < 0.01) and 0.18 (*p* < 0.01) more lambs than the ewes carrying genotype *TT*, respectively.

In Hu sheep, the ewes with genotypes *BB* and *B+* had 0.74 and 0.59 had more lambs than those with genotype *++*, respectively. While the ewes carrying genotype *G+* had 0.52 (*p* < 0.05) more lambs than the *++* genotype ones. The ewes carrying genotype *CC* had 0.80 (*p* < 0.01) and 0.54 (*p* < 0.01) had more lambs when compared to the ewes carrying genotypes *TT* and *TC*, respectively.

**Table 1:** Allele and genotype frequencies of the ovine *BMPR-IB*, *BMP-15* and *FSHR* genes in experimental populations.

Gene	Total Population					Small Tail Han Sheep					Hu Sheep				
	Genotype Frequency			Allele Frequency		Genotype Frequency			Allele Frequency		Genotype Frequency			Allele Frequency	
*BMPR-IB*	*BB*	*B+*	*++*	*B*	*+*	*BB*	*B+*	*++*	*B*	*+*	*BB*	*B+*	*++*	*B*	*+*
	0.09	0.89	0.02	0.53	0.47	0.10	0.88	0.02	0.54	0.46	0.08	0.90	0.02	0.53	0.47
*BMP-15*	*GG*	*G+*	*++*	*G*	*+*	*GG*	*G+*	*++*	*G*	*+*	*GG*	*G+*	*++*	*G*	*+*
	0	0.29	0.71	0.15	0.85	0	0.4	0.6	0.20	0.80	0	0.17	0.83	0.08	0.92
*FSHR*	*CC*	*TC*	*TT*	*C*	*T*	*CC*	*TC*	*TT*	*C*	*T*	*CC*	*TC*	*TT*	*C*	*T*
	0.02	0.61	0.37	0.33	0.67	0.02	0.42	0.56	0.24	0.76	0.02	0.83	0.15	0.44	0.56

*B* means *FecB* mutation; *G* means *FecX^G^* mutation; + means wild-type; *C* means *FSHR* g.47C>T mutation; *T* means wild-type.

**Table 2:** Association results between the genotypes of the ovine *BMPR-IB*, *BMP-15* and *FSHR* genes and litter size.

Gene	Genotype ^1^	Total Population		Small Tail Han Sheep		Hu Sheep	
		No. of Ewes	Litter Size	No. of Ewes	Litter Size	No. of Ewes	Litter Size
*BMPR-IB*	*BB*	142	1.95 ± 0.070 ^A^	85	2.06 ± 0.078 ^A^	57	1.89 ± 0.092 ^A^
	*B+*	1450	1.75 ± 0.020 ^B^	765	1.78 ± 0.026 ^B^	685	1.74 ± 0.026 ^B^
	*++*	38	1.17 ± 0.115 ^C^	19	1.21 ± 0.166 ^C^	19	1.15 ± 0.150 ^C^
*BMP-15*	*G+*	479	1.96 ± 0.035 ^a^	351	1.91 ± 0.039	128	2.18 ± 0.063 ^a^
	*++*	1151	1.66 ± 0.019 ^b^	518	1.71 ± 0.032	633	1.66 ± 0.028 ^b^
*FSHR*	*CC*	39	2.31 ± 0.124 ^Aa^	21	2.33 ± 0.157 ^Aa^	18	2.29 ± 0.158 ^A^
	*TC*	1000	1.79 ± 0.023 ^Bb^	368	1.88 ± 0.038 ^Ab^	632	1.75 ± 0.028 ^B^
	*TT*	591	1.56 ± 0.050 ^Bc^	480	1.70 ± 0.033 ^Bc^	111	1.49 ± 0.068 ^B^

The numbers in the table are the LSMEAN ± standard error of phenotypic value. The different superscript letters in the same column for each gene in lowercase represent significant level at *p* < 0.05, which letters in uppercase represent significant level at *p* < 0.01, and the same letter represents no significant difference (*p* > 0.05). ^1^ *B* means *FecB* mutation; *G* means *FecX^G^* mutation; + means wild-type; *C* means *FSHR* g.47C>T mutation; *T* means wild-type.

**Table 3:** Combined effect analysis of two genes (*BMPR-IB/BMP-15*, *BMPR-IB/FSHR* and *BMP-15*/*FSHR*) on litter size.

Gene	Genotype	Total Population		Small Tail Han Sheep		Hu Sheep	
		No. of Ewes	Litter Size	No. of Ewes	Litter Size	No. of Ewes	Litter Size
*BMPR-IB/BMP-15*	*++/++*	29	1.17 ± 0.071 ^Dd^	13	1.15 ± 0.104 ^Bc^	16	1.19 ± 0.101 ^Bbc^
	*++/G+*	9	1.22 ± 0.147 ^Dcd^	6	1.33 ± 0.211 ^Bbc^	3	1.00 ± 0.000 ^Bc^
	*B+/++*	1029	1.66 ± 0.020 ^BCbc^	450	1.71 ± 0.033 ^ABbc^	579	1.63 ± 0.025 ^ABabc^
	*BB/++*	93	1.82 ± 0.073 ^ABb^	55	1.84 ± 0.089 ^ABab^	38	1.79 ± 0.126 ^ABab^
	*B+/G+*	412	1.93 ± 0.036 ^ABab^	315	1.87 ± 0.041 ^ABab^	106	2.13 ± 0.068 ^Aa^
	*BB/G+*	49	2.35 ± 0.129 ^Aa^	30	2.47 ± 0.157 ^Aa^	19	2.16 ± 0.220 ^Aa^
*BMPR-IB/FSHR*	++/*TC*	23	1.17 ± 0.081 ^Dd^	8	1.13 ± 0.125 ^Bd^	15	1.20 ± 0.107
	++/*TT*	15	1.20 ± 0.107 ^Dcd^	11	1.27 ± 0.141 ^Bcd^	4	1.00 ± 0.000
	*B*+/*TT*	518	1.67 ± 0.030 ^BCbcd^	431	1.69 ± 0.033 ^Bbcd^	87	1.57 ± 0.069
	*B*+/*TC*	896	1.76 ± 0.022 ^BCbcd^	315	1.86 ± 0.042 ^ABbcd^	581	1.70 ± 0.026
	*BB*/*TT*	58	1.91 ± 0.099 ^BCbc^	38	1.89 ± 0.112 ^ABbcd^	20	1.95 ± 0.198
	*BB*/*TC*	81	2.02 ± 0.093 ^BCb^	45	2.16 ± 0.123 ^ABabc^	36	1.86 ± 0.139
	*B*+/*CC*	36	2.31 ± 0.125 ^ABab^	19	2.26 ± 0.168 ^ABab^	17	2.35 ± 0.191
	*BB*/*CC*	3	3.000 ± 0.577 ^Aa^	2	3.00 ± 1.000 ^Aa^	1	3.00
*BMP-15/FSHR*	++/*TT*	425	1.63 ± 0.032 ^Cc^	340	1.66 ± 0.036 ^Bc^	85	1.51 ± 0.072 ^Cd^
	++/*TC*	707	1.67 ± 0.024 ^Cc^	169	1.78 ± 0.058 ^ABbc^	538	1.63 ± 0.025 ^BCcd^
	*G*+/*TT*	166	1.81 ± 0.055 ^BCc^	140	1.78 ± 0.60 ^ABbc^	26	2.00 ± 0.136 ^ABCbcd^
	*G*+/*TC*	293	2.01 ± 0.045 ^BCbc^	199	1.97 ± 0.055 ^ABabc^	94	2.10 ± 0.080 ^ABCabc^
	++/*CC*	19	2.21 ± 0.181 ^ABab^	9	2.22 ± 0.222 ^ABab^	10	2.20 ± 0.291 ^ABab^
	*G*+/*CC*	20	2.50 ± 0.170 ^Aa^	12	2.42 ± 0.260 ^Aa^	8	2.63 ± 0.183 ^Aa^

The numbers in the table are the LSMEAN ± standard error of phenotypic value. The different superscript letters in the same column for each gene in lowercase represent significant level at *p* < 0.05, which letters in uppercase represent significant level at *p* < 0.01, and the same letter represents no significant difference (*p* *>* 0.05).^1^ *B* means *FecB* mutation; *G* means *FecX^G^* mutation; + means wild-type; *C* means *FSHR* g.47C>T mutation; *T* means wild-type.

**Table 4:** Combined effect analysis of *BMPR-IB*, *BMP-15* and *FSHR* genes on litter size.

Gene	Genotype	Total Population		Small Tail Sheep		Hu Sheep	
		No. of Ewes	Litter Size	No. of Ewes	Litter Size	No. of Ewes	Litter Size
*BMPR-IB/BMP-15/FSHR*	++/*G*+/*TC*	5	1.00 ± 0.000 ^Ee^	2	1.00 ± 0.000	3	1.00 ± 0.000
	++/++/*TT*	11	1.09 ± 0.091 ^D^^Ede^	7	1.14 ± 0.143	4	1.00 ± 0.000
	++/++/*TC*	18	1.22 ± 0.101 ^CD^^Ec^^de^	6	1.17 ± 0.167	12	1.25 ± 0.131
	++/*G*+/*TT*	4	1.50 ± 0.289 ^BCD^^Ebc^^de^	4	1.50 ± 0.289	0	-
	*B*+/++/*TT*	378	1.64 ± 0.034 ^BCD^^Ebc^^de^	307	1.67 ± 0.038	71	1.51 ± 0.075
	*B*+/++/*TC*	633	1.66 ± 0.025 ^BCD^^Ebc^^de^	135	1.78 ± 0.066	498	1.63 ± 0.026
	*BB*/++/*TT*	36	1.75 ± 0.122 ^BDC^^Ebc^^de^	26	1.77 ± 0.128	10	1.70 ± 0.300
	*B*+/*G*+/*TT*	140	1.76 ± 0.059 ^BCD^^Ebc^^de^	124	1.75 ± 0.063	16	1.88 ± 0.155
	*BB*/++/*TC*	56	1.86 ± 0.093 ^BCD^^Ebc^^de^	28	1.89 ± 0.130	28	1.82 ± 0.137
	*B*+/*G*+/*TC*	263	1.99 ± 0.045 ^BCD^^Ebc^^d^	180	1.92 ± 0.055	83	2.14 ± 0.079
	*BB*/*G*+/*TT*	22	2.18 ± 0.156 ^BCDbc^	12	2.17 ± 0.207	10	2.20 ± 0.249
	*B*+/++/*CC*	18	2.22 ± 0.191 ^BCb^	8	2.25 ± 0.250	10	2.20 ± 0.291
	*B*+/*G*+/*CC*	18	2.39 ± 0.164 ^Bb^	11	2.27 ± 0.237	7	2.57 ± 0.202
	*BB*/*G*+/*TC*	25	2.40 ± 0.200 ^Bb^	17	2.59 ± 0.211	8	2.00 ± 0.423
	*BB*/*G*+/*CC*	2	3.50 ± 0.500 ^Aa^	1	4.00	1	3.00

The different superscript letters in the same column for each gene in lowercase represent significant level at *p* < 0.05, which letters in uppercase represent significant level at *p* < 0.01, and the same letter represents no significant difference (*p* > 0.05). *B* means *FecB* mutation; *G* means *FecX^G^* mutation; + means wild-type; *C* means *FSHR* g.47C>T mutation; *T* means wild-type.

### 2.3. Combined Effect Analysis of BMPR-IB, BMP-15 and FSHR Genes on Litter Size

Highly significant interactions were observed if we randomly combined two of three genes studied and all the three genes. The combined effect of two genes (*BMPR-IB/BMP-15*, *BMPR-IB/FSHR* and *BMP-15/FSHR*) on litter size is presented in Table 3. For *BMPR-IB/BMP-15*, the ewes with *BB*/*G+* genotype had the largest litter size and with the *++*/*++* genotype having the lowest litter size among all the six genotypes. The effect of the *BMPR-IB* gene mutation was greater than that of the *BMP-15* gene mutation on litter size in this population. For *BMPR-IB/FSHR*, the ewes with *BB/CC* genotype had greater litter size than those with other genotypes. The effect of the *BMPR-IB* gene mutation was greater than that of the *FSHR* gene mutation on litter size. For *BMP-15*/*FSHR*, the ewes with *G+/CC* genotype had greater litter size than those with other genotypes. The effect of the *FSHR* gene mutation was greater than that of the *BMP-15* gene mutation on litter size. Therefore, the ewes carrying mutations in both the *BMPR-IB* and *FSHR* genes had greater litter size than the other two genes combinations, and the effect of the *BMPR-IB* mutation was the greatest among these three genes.

The combined effect analysis of three genes (*BMPR-IB/BMP-15/FSHR*) on litter size is presented in Table 4. The *BB/G+/CC* genotype had significantly greater contribution on litter size than any other genotypes.

## 3. Discussion

In the present study, we selected the ovine *BMPR-IB*, *BMP-15* and *FSHR* as candidate genes to analyze the effect of single-marker and multi-marker on litter size. The *FecB* gene is crucial in the regulation of prolificacy phenotype in sheep [1,2]. Several studies indicated that ewes carrying *FecB*-mutation have significantly higher ovulation rates if compared with their wild-type contemporaries [1,2,14]. In this study, the *FecB*-mutation was found in Small Tailed Han sheep and Hu sheep, and was significantly associated with litter size, which is consistent with previous reports [15,16,17,18].

Ovine *BMP-15* gene plays a vital role in growth and differentiation of early ovarian follicles [19,20,21,22]. In Inverdale and Hanna sheep, the c.718C>T mutation of the *BMP-15* gene has been reported to show an increased ovulation rate under heterozygous conditions, and homozygotes are otherwise infertile [17]. Chu *et al.* (2005), Wang *et al.* (2005) and Davis *et al.* (2006) failed to detect the *BMP-15* (FecXI) mutation in Hu sheep [17,23,24], but a *BMP-15* (FecXG) was identified in the Small Tailed Han Sheep by Chu *et al.* [15]. In the present study, the *BMP-15* (FecXG) mutation was detected in both Small Tailed Han and Hu sheep breeds. We also found that the c.718C>T mutation of the *BMP-15* gene was significantly association with litter size, similar with previous studies in the Inverdale and Hanna sheep [17]. Interestingly, there were no homozygotes (*GG* genotype) detected in Small Tailed Han sheep (*n* = 869) and Hu sheep (*n* = 761). Chu *et al.* also reported the absence of *GG* genotype in Small Tailed Han sheep [15]. There are two potential reasons for the lack of *GG* genotype ewes in the population, one simple explanation is that *GG* ewes did not exist in our population, another reason is that the *GG* ewes may have existed in our population, but we selected the ewes with litter size records, and the infertile *GG* ewes were excluded in this study. About 29% of ewes in the population are *G+* genotype and mating occurred under a random model; we believe *GG* genotype ewes should be generated under this model and therefore the *GG* genotype ewes should be detected in the infertile group. Mating of the *G+* genotype rams and *G+* genotype ewes can help verify this speculation.

Numerous reports have shown that the *FSHR* gene plays a key role in animal reproduction [25,26,27]. Chu *et al.* found two mutation (g.681T>C and g.629C>T) in the 5' flanking region of the *FSHR* gene in Hu sheep and three novel mutations (g.200G>A, g.197G>A and g.98T>C) in Small Tail Han Sheep [12]. In our previous study, a novel SNP (g.47C>T) was found in the 5' flanking region of the *FSHR* gene in the Small Tailed Han sheep and Hu sheep [14]. This SNP was significantly associated with litter size. Therefore, the ovine *FSHR* gene could be selected as a candidate gene for improving litter size traits in sheep husbandry.

Interestingly, several groups have reported the multi-marker combination effect on litter size in sheep. Chu *et al.* reported that the Small Tailed Han ewes carried *BB/G+* genotype (*BMPR-IB* and *BMP-15*) showed more litter size than those with either mutation alone [15]. Individuals in Cambridge and Belclare breeds with mutations in both the *GDF9* and *BMP15* genes were found to be associated with greater ovulation rate than those with either single mutation [6]. In the present study, mutations in both the *BMPR-IB* and *BMP-15* genes were also detected in Hu sheep and Small Tailed Han sheep and we also found a third mutations (*FSHR* g.47C>T) in these two breeds. The single marker-trait association analysis revealed that each mutation in ovine *BMPR-IB*, *BMP-15* and *FSHR* genes was significantly associated with litter size in this population, and multi-marker analysis showed that individuals with the *BB/G+/CC* genotype had more lambs than those with only one predominant genotype, indicating that multiple markers may have a greater effect on contributing to the litter size in sheep and that the *BB/G+/CC* genotype combinations of *BMPR-IB*, *BMP-15* and *FSHR* genes was considered as the superior genotype.

## 4. Experimental Section

### 4.1. Ethics Statement

The experimental procedures were performed according to protocols approved by the Biological Studies Animal Care and Use Committee of Gansu Province, China. All efforts were made to minimize any discomfort during blood collection.

### 4.2. Experimental Population

A total of 1630 ewes aged from 12 to 30 months were collected from Gansu Zhongtian Sheep Ltd., including 869 Small Tail Han Sheep and 761 Hu sheep. All the sheep were in the artificial insemination system and raised in the same managed conditions. The litter size data for ewes was from the first or second parity (Table 5).

**Table 5:** Experimental population structure and litter size phenotypic value.

Breed	No.	Litter Size
Hu Sheep	761	1.706 ± 0.024 ^a^
Small Tail Han Sheep	869	1.791 ± 0.025 ^a^
Total	1630	1.752 ± 0.017

The numbers in the table are the LSMEAN ± standard error of the litter size. The same letter represents no significant difference (*p* > 0.05).

### 4.3. DNA Extraction and Genotyping

Genomic DNA was extracted from the venous jugular blood samples (5 mL per ewes) by the phenol-chloroform method, then dissolved in TE buffer solution (10 mM Tris-HCl and 1 mM EDTA, pH 8.0), and kept at −20 °C.

The polymorphisms were genotyped by PCR-RFLP. The primers and restriction enzymes used in the genotyping analysis are listed in Table 6. The information of the primers of *BMPR-IB*, *BMP-15* and *FSHR* are shown elsewhere [1,6,13]. The PCR was performed in a volume of 10 μL, containing 10× PCR buffer, 0.15 μM primer, 35 μM of dNTP, and 20 ng of genomic DNA, 0.5 U Taq DNA Polymerase (TransGen Biotech, Beijing, China). The PCR was performed as below: 5 min at 94 °C, followed by 35 cycles for 30 s at 94 °C, 30 s at 58~63 °C, 25 s at 72 °C and a final extension of 5 min at 72 °C. Five μL of each PCR product was digested with 3 U restriction endonuclease overnight at 37 °C, then the different sizes were separated on a 3% agarose gel, subsequently stained by GelRed. PCR fragments from different genotypes were cloned and sequenced for validation.

**Table 6:** Primers and restriction endonucleases.

Gene	Primer Sequences (5'-3')	Tm (°C)	PCR Product Size (bp)	Restriction Endonuclease	Citation
*BMPR-IB*-F	GTCGCTATGGGGAAGTTTGGATG	59	140	*Ava* II	[1]
*BMPR-IB*-R	CAAGATGTTTTCATGCCTCATCAACACGGTC				
*BMP-15*-F	CACTGTCTTCTTGTTACTGTATTTCAATGAGAC	63	141	*Hinf* I	[6]
*BMP-15*-R	GATGCAATACTGCCTGCTTG				
*FSHR*-F	CGTATCTTTCCACGCCCTCT	58	244	*BsiE* I	[14]
*FSHR*-R	CCATCCACCCGATTGCTT				

### 4.4. Statistical Analysis

The association analysis between single marker and litter size was performed by GLM (General liner model) procedure in the SAS software package (SAS Inst. Inc., Cary, NC, USA). The linear model was as follows:

Y_ijl_ = μ + G_i_ + B_j_ + S_l_ + ε_ijl_

where Y_ijl_ was the ijl traits’ observation value; μ was the mean; G_i_ was the effect of the ith genotypes; B_j_ was the effect of jth breeding; S_l_ was the effect within season and ε_ijl_ was residual corresponding to the traits observation value with var (ε) = Iσ_e_^2^.

The model of association analysis between multiple markers and litter size was as follows:

Y = μ + SNP1 + SNP2 + SNP1 × SNP2 + B_j_ + S_l_ + C_m_ + ε_jlm_

and

Y = μ + SNP1 + SNP2 + SNP3 + SNP1 × SNP2 + SNP1 × SNP3 + SNP2 × SNP3+ SNP1 × SNP2 × SNP3 + B_j_ + S_l_ + Combination_m_ + ε_jlm_

where μ was the traits’ mean; SNP1, SNP2 and SNP3 were the effect of the genotypes; SNP1 × SNP2, SNP1 × SNP3 and SNP2 × SNP3 were combined effects of double genes; SNP1 × SNP2 × SNP3 was combined effect of three genes; B_j_ was the effect of jth breeding; S_l_ was the effect within season; Combination_m_ was the effect of combination and ε_jlm_ was residual corresponding to the traits observation value with var (ε) = Iσ_e_^2^. *p* ≤ 0.05 was considered as the statistically significant criterion.

## 5. Conclusions

In summary, our present study indicated that the Small Tailed Han sheep and Hu sheep carried three polymorphisms (*FecB*, *FecG* and *FSHR* g.47C>T) associated with litter size. The ovine *BMPR-IB*, *BMP-15* and *FSHR* genes have a combined effect on litter size in Small Tailed Han sheep and Hu sheep. Using *BMPR-IB*, *BMP-15* and *FSHR* genes as genetic markers for multi-gene pyramiding can provide a way to improve litter size and shorten the breeding process of highly prolific sheep.

## References

[1] Wilson T., Wu X.Y., Juengel J.L., Ross I.K., Lumsden J.M., Lord E.A., Dodds K.G., Walling G.A., McEwan J.C., OʼConnell A.R. (2001). Highly prolific Booroola sheep have a mutation in the intracellular kinase domain of bone morphogenetic protein IB receptor (ALK-6) that is expressed in both oocytes and granulosa cells. Biol. Reprod..

[2] Mulsant P., Lecerf F., Fabre S., Schibler L., Monget P., Lanneluc I., Pisselet C., Riquet J., Monniaux D., Callebaut I. (2001). Mutation in bone morphogenetic protein receptor-IB is associated with increased ovulation rate in Booroola Merino ewes. Proc. Natl. Acad. Sci. USA.

[3] Souza C.J., MacDougall C., MacDougall C., Campbell B.K., McNeilly A.S., Baird D.T. (2001). The Booroola (FecB) phenotype is associated with a mutation in the bone morphogenetic receptor type 1 B (*BMPR1B*) gene. J. Endocrinol..

[4] Galloway S.M., McNatty K.P., Cambridge L.M., Laitinen M.P., Juengel J.L., Jokiranta T.S., McLaren R.J., Luiro K., Dodds K.G., Montgomery G.W. (2000). Mutations in an oocyte-derived growth factor gene (BMP-15) cause increased ovulation rate and infertility in a dosage-sensitive manner. Nat. Genet..

[5] Crawford J.L., Heath D.A., Reader K.L., Quirke L.D., Hudson N.L., Juengel J.L., McNatty K.P. (2011). Oocytes in sheep homozygous for a mutation in bone morphogenetic protein receptor 1B express lower mRNA levels of bone morphogenetic protein 15 but not growth differentiation factor 9. Reproduction.

[6] Hanrahan J.P., Gregan S.M., Mulsant P., Mullen M., Davis G.H., Powell R., Galloway S.M. (2004). Mutations in the genes for oocyte-derived growth factors GDF9 and BMP-15 are associated with both increased ovulation rate and sterility in Cambridge and Belclare sheep (*Ovis aries*). Biol. Reprod..

[7] Sprengel R., Braun T., Nikolics K., Segaloff D.L., Seeburg P.H. (1990). The Testicular Receptor for Follicle-Stimulating-Hormone—Structure and functional expression of cloned cDNA. Mol. Endocrinol..

[8] Gharib S.D., Wierman M.E., Shupnik M.A., Chin W.W. (1990). Molecular-biology of the pituitary gonadotropins. Endocr. Rev..

[9] Tisdall D.J., Watanabe K., Hudson N.L., Smith P., Mcnatty K.P. (1995). Fsh Receptor gene-expression during ovarian follicle development in sheep. J. Mol. Endocrinol..

[10] Sairam M.R., Subbarayan V.S.R. (1997). Characterization of the 5' flanking region and potential control elements of the ovine follitropin receptor gene. Mol. Reprod. Dev..

[11] Liu S.F., Du L.X., Wang A.H. (2006). Biological characteristics of the 5' regulatory region of *FSHR* gene in sheep. Yi Chuan.

[12] Chu M.X., Guo X.H., Feng C.J., Li Y., Huang D.W., Feng T., Cao G.L., Fang L., di R., Tang Q.Q. (2012). Polymorphism of 5' regulatory region of ovine *FSHR* gene and its association with litter size in Small Tail Han sheep. Mol. Biol. Rep..

[13] Zhao Y.Z. (2003). Zhong Guo Yang Yang Xue.

[14] Pan X., Liu S., Li F., Wang W., Li C., Ma Y., Li T. (2014). Molecular characterization, expression profiles of the ovine *FSHR* gene and its association with litter size. Mol. Biol. Rep..

[15] Chu M.X., Liu Z.H., Jiao C.L., He Y.Q., Fang L., Ye S.C., Chen G.H., Wang J.Y. (2007). Mutations in *BMPR-IB* and *BMP-15* genes are associated with litter size in Small Tailed Han sheep(*Ovis aries*). J. Anim. Sci..

[16] Davis G.H., Galloway S.M., Ross I.K., Gregan S.M., Ward J., Nimbkar B.V., Ghalsasi P.M., Nimbkar C., Gray G.D., Subandriyo (2002). DNA tests in prolific sheep from eight countries provide new evidence on origin of the Booroola (FecB) mutation. Biol. Reprod..

[17] Davis G.H., Balakrishnan L., Ross I.K., Wilson T., Galloway S.M., Lumsden B.M., Hanrahan J.P., Mullen M., Mao X.Z., Wang G.L. (2006). Investigation of the Booroola (FecB) and Inverdale (FecX(I)) mutations in 21 prolific breeds and strains of sheep sampled in 13 countries. Anim. Reprod. Sci..

[18] Guan F., Liu S.R., Shi G.Q., Yang L.G. (2007). Polymorphism of *FecB* gene in nine sheep breeds or strains and its effects on litter size, lamb growth and development. Anim. Reprod. Sci..

[19] McNatty K.P., Moore L.G., Hudson N.L., Quirke L.D., Lawrence S.B., Reader K., Hanrahan J.P., Smith P., Groome N.P., Laitinen M. (2004). The oocyte and its role in regulating ovulation rate: A new paradigm in reproductive biology. Reproduction.

[20] Shimasaki S., Moore R.K., Otsuka F., Erickson G.F. (2004). The bone morphogenetic protein system in mammalian reproduction. Endocr. Rev..

[21] Juengel J.L., McNatty K.P. (2005). The role of proteins of the transforming growth factor-β superfamily in the intraovarian regulation of follicular development. Hum. Reprod. Update.

[22] Fabre S., Pierre A., Mulsant P., Bodin L., di Pasquale E., Persani L., Monget P., Monniaux D. (2006). Regulation of ovulation rate in mammals: Contribution of sheep genetic models. Reprod. Biol. Endocr..

[23] Chu M.X., Sang L.H., Wang J.Y., Fang L., Ye S.C. (2005). Study on *BMP-15* and *GDF9* as candidate genes for prolificacy of Small Tail Han sheep. Yi Chuan.

[24] Wang Q.G., Zhong F.G., Li H., Wamg X.H., Liu S.R., Chen X.J., Gan S.Q. (2005). Detection of major gene on litter size in sheep. Yi Chuan.

[25] Banerjee A.A., Dupakuntla M., Pathak B.R., Mahale S. (2015). FSH receptor specific residues L501 and I505 in extracellular loop 2 are essential for its function. J. Mol. Endocrinol..

[26] Rivera O.E., Varayoud J., Rodriguez H.A., Santamaria C.G., Bosquiazzo V.L., Osti M., Belmonte N.M., Munoz-de-Toro M.M., Luque E.H. (2015). Neonatal exposure to xenoestrogens impairs the ovarian response to gonadotropin treatment in lambs. Reproduction.

[27] Zhang R., Zhang S., Zhu X., Zhou Y., Wu X. (2015). Follicle-stimulating hormone receptor (FSHR) in Chinese alligator, Alligator sinensis: Molecular characterization, tissue distribution and mRNA expression changes during the female reproductive cycle. Anim. Reprod. Sci..

